# Blood biomarkers for mild traumatic brain injury: a selective review of unresolved issues

**DOI:** 10.1186/s40364-021-00325-5

**Published:** 2021-09-16

**Authors:** Daniel B. Hier, Tayo Obafemi-Ajayi, Matthew S. Thimgan, Gayla R. Olbricht, Sima Azizi, Blaine Allen, Bassam A. Hadi, Donald C. Wunsch

**Affiliations:** 1 Department of Electrical and Computer Engineering, Missouri University of Science and Technology, Rolla, MO 65401 USA; 2 Cooperative Engineering Program, Missouri State University, Springfield, MO 65897 United States; 3 Department of Biological Sciences, Missouri University of Science and Technology, Rolla, MO 65409 United States; 4 Department of Mathematics and Statistics, Missouri University of Science and Technology, Rolla, MO 65409 United States; 5 Department of Surgery, Mercy Hospital, St. Louis MO, Missouri, MO 63141 United States; 6 National Science Foundation, ECCS Division, Virginia, 22314 USA

**Keywords:** NF-L, UCH-L1, GFAP, Mild traumatic brain injury, Kinetics, S100B, Blood biomarkers, Tau, Concussion, Return to sport, CT scan

## Abstract

**Background:**

The use of blood biomarkers after mild traumatic brain injury (mTBI) has been widely studied. We have identified eight unresolved issues related to the use of five commonly investigated blood biomarkers: neurofilament light chain, ubiquitin carboxy-terminal hydrolase-L1, tau, S100B, and glial acidic fibrillary protein. We conducted a focused literature review of unresolved issues in three areas: mode of entry into and exit from the blood, kinetics of blood biomarkers in the blood, and predictive capacity of the blood biomarkers after mTBI.

**Findings:**

Although a disruption of the blood brain barrier has been demonstrated in mild and severe traumatic brain injury, biomarkers can enter the blood through pathways that do not require a breach in this barrier. A definitive accounting for the pathways that biomarkers follow from the brain to the blood after mTBI has not been performed. Although preliminary investigations of blood biomarkers kinetics after TBI are available, our current knowledge is incomplete and definitive studies are needed. Optimal sampling times for biomarkers after mTBI have not been established. Kinetic models of blood biomarkers can be informative, but more precise estimates of kinetic parameters are needed. Confounding factors for blood biomarker levels have been identified, but corrections for these factors are not routinely made. Little evidence has emerged to date to suggest that blood biomarker levels correlate with clinical measures of mTBI severity. The significance of elevated biomarker levels thirty or more days following mTBI is uncertain. Blood biomarkers have shown a modest but not definitive ability to distinguish concussed from non-concussed subjects, to detect sub-concussive hits to the head, and to predict recovery from mTBI. Blood biomarkers have performed best at distinguishing CT scan positive from CT scan negative subjects after mTBI.

## Background

Mild traumatic brain injury (mTBI), also known as concussion, has been defined as a minor head injury with a Glasgow Coma Scale score of 13 to 15. Loss of consciousness and post traumatic amnesia occur variably. The CT scan and MRI may be normal or show minor abnormalities. A variety of protein macromolecules and smaller molecules detectable in the blood and cerebrospinal fluid have been investigated as biomarkers for mTBI [1–13]. This review focuses on five of the most commonly studied markers for mTBI: glial fibrillary acidic protein (GFAP), neurofilament light chain protein (NF-L), ubiquitin C-terminal hydrolase-L1 (UCH-L1), tau, and S100B. Since these five protein biomarkers have been extensively reviewed, we briefly highlight some of their important features as a context for this review. Glial fibrillary acidic protein, a 50 kDa protein, is the primary component of intermediate filaments in astrocytes [5]. GFAP monomers homo-polymerize to form the intermediate filament cytoskeleton of astrocytes [14, 15]. GFAP is upregulated during astrogliosis and is likely released from astrocytes after traumatic brain injury [6]. Levels of GFAP are elevated in both the cerebrospinal fluid and blood after TBI [16, 17]. Neurofilament protein comes in three forms: a 200 kDa heavy chain (NF-H), a 150 kDa medium-chain (NF-M), and a 68 kDa light chain (NF-L). The backbones of 10 nm diameter neurofilaments in neurons are assembled by the polymerization of neurofilament light chains and the side arms are made from neurofilament medium and heavy chains [18, 19]. Mature neurofilaments provide a stable framework within the axons of neurons. NF-L protein has a slow turnover in axons with an estimated intracellular half-life of 3 weeks [18, 20]. NF-L levels are increased in both the blood and cerebrospinal fluid after TBI [21]. Ubiquitin C-terminal hydrolase-L1 is a 25 kDa enzyme that is highly expressed in neurons. It is involved in ubiquitination and de-ubiquitination of proteins allocated for catabolism. Blood and cerebrospinal fluid levels of UCH-L1 are elevated after TBI [22].

Tau is a protein involved in the stabilization of microtubules in the axons of neurons in both the central and peripheral nervous system. In the central nervous system, tau has six isoforms (A-F) with molecular weights ranging from 33 kDa to 46 kDa [23]. Tau has been measured after TBI in a variety of forms, including cleaved-tau (tau-C), phosphorylated-tau (tau-P), and total tau (tau-T). Although elevated tau levels indicate neuronal injury, tau is released into the interstitial fluid by healthy neurons [24]. Intracellular turnover of tau is slow, with an estimated half-life of 19-30 days [24]. Tau is elevated in the plasma and cerebrospinal fluid after TBI [25]. S100B (formerly S100- *β*) is a low-molecular weight 10 kDa calcium-binding protein expressed in glia and Schwann cells. Blood levels of S100B may be elevated after trauma without head injury [7]. Immediately after TBI, extracranial sources of S100B may be significant [26]. Elimination of S100B from the blood is rapid with a half-life between 0.5 and 2 hrs [26–28]. After TBI, levels in the cerebrospinal fluid may be up to 100 times higher than serum levels [26].

### Selection of issues for focused review

Despite much investigation of blood biomarkers in the settings of mild, moderate, and severe brain injury, some issues regarding their use remain unresolved [4–12]. McDonald et al. [13] have recently emphasized that known unknowns exist for the use of blood biomarkers for mTBI including the mechanism by which biomarkers gain entry to the blood (via blood brain barrier or glymphatic system), biomarker kinetics including rates of absorption and elimination, and analytic issues (including obtaining, processing, and analyzing blood samples). This review focuses on eight unresolved issues related to the use of blood biomarkers to diagnose and manage mTBI

1. How do biomarkers enter and exit the blood?
2. What are the kinetics of blood biomarkers?
3. What is the optimal sampling time for blood biomarkers?
4. How long do the blood biomarker levels remain elevated?
5. What are the confounding factors for blood biomarker levels?
6. Can blood biomarkers differentiate between subjects with concussions or sub-concussive hits to the head from healthy controls?
7. Can blood biomarkers predict CT scan positivity?
8. Can blood biomarkers predict outcome or severity of mTBI?

This list of unresolved issues is selective and is not comprehensive. Most of the literature discussed in this paper stems from the intersection of the searches in *Ovid MEDLINE and Epub Ahead of Print (1946 to February 5, 2021)* for the keyword **traumatic brain injuries** (15,999) and the search terms **UCH-L1** OR **GFAP** OR **tau proteins** OR **S100 proteins** OR **neurofilament proteins** (54,631) which yielded 406 articles. We added 202 articles with the search terms **glymphatic system** OR **periarterial drainage**. We added 195 articles from ad hoc searches. We reviewed the titles and abstracts of 803 full-text publications to select the 140 articles retained for this review.

## Review findings

### How do biomarkers enter and exit the blood after mTBI?

**Fig. 1:**
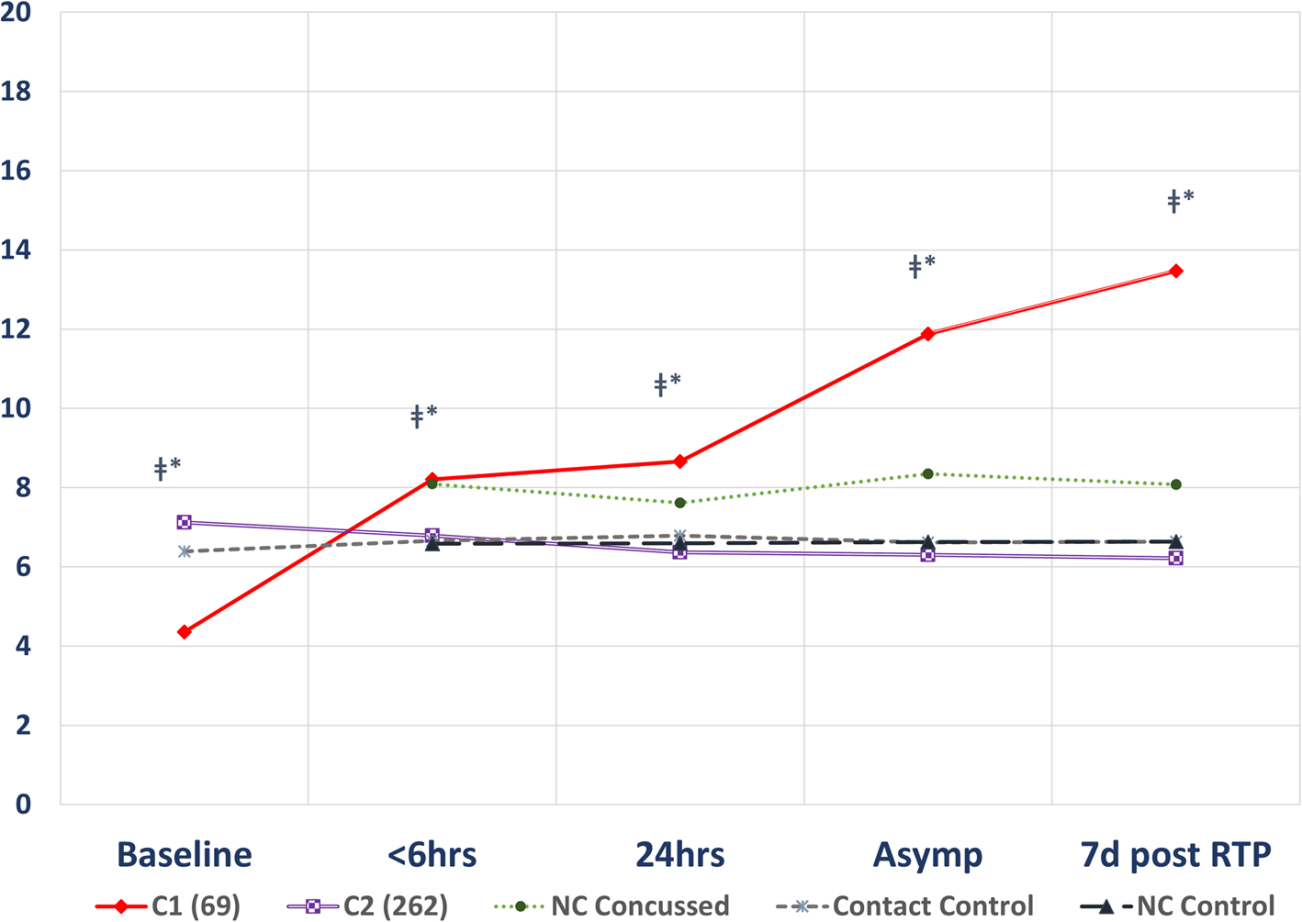
Cluster trajectories for NF-L biomarker for concussed contact sport subjects formed two clusters C1 and C2. C1 (red line) showed a robust rise in NF-L after concussion whereas C2 (purple line) showed a modest elevation in NF-L not much different from the non-contact sport concussed, contact sport controls, and not-contact sport controls. C1 and C2 differed at all time points (p <0.05). Reproduced from [29] with permission. Original data from CARE study [30]

After mTBI, axonal shearing and cellular disruption cause the release of biomarkers from neurons and astrocytes [1, 8]. NF-L, UCH-L1, and tau are released from neurons; GFAP and S100B are released from astrocytes [8]. These biomarkers are released into the surrounding interstitial fluid. A dose-response relationship for the release of biomarkers has not been established, i.e. there no proven linear or non-linear relationship between the amount of biomarker released into the interstitial fluid and the force of the impact to the head. Bui et al. [29] examined the modality of blood biomarker levels after mTBI in a dataset of concussed athletes from the Concussion Assessment, Research, and Education Consortium Study (CARE) study [30, 31]. (Data is available via the Federal Interagency Traumatic Brain Injury Research (FITBIR) [32] repository to approved investigators.). Based on a test for modality, we found that biomarker levels after concussion were multi-modal rather that than uni-modal [29]. When subjects were clustered by biomarker trajectory after mTBI, some clusters showed a robust rise in biomarker levels while others showed a modest or minimal rise in biomarker levels [29] (Fig. 1).

With a volume of approximately 1200 ml, the human brain is tightly packed with cellular elements (neurons, astrocytes, oligodendrocytes and their myelin, and microglia). About 20% of the brain is extracellular space [33]; filled with an extracellular matrix and approximately 120 ml of interstitial fluid. The interstitial fluid brings nutrients to the neurons and glia of the brain and removes waste products. Proteins move through the interstitial fluid by diffusion or more rapidly by convection [33–35]. The extracellular space is highly confined with narrow passages that are 38-64 nm diameter [33]. Given the large size of some protein biomarkers (Table 1), these tight passages can slow the diffusion of biomarkers. Movement in these tight passages is by hindered diffusion and larger molecules are slowed more than smaller molecules [33, 37]

**Table 1:** Some Properties of Proteins Investigated as Biomarkers for mTBI

Biomarker	MW†	Residues†	Role	Localization	Cell
S100B	10.7	92	regulatory	cytoplasm	astrocyte
UCH-L1	24.8	223	enzymatic	cytoplasm	neuron
tau isoform A§	32.9	316	structural	axon	neuron
tau isoform F§	45.8	441	structural	axon	neuron
GFAP	49.9	543	structural	cytoplasm	astrocyte
NF-L	61.5	304	structural	axon	neuron

^§^Human CNS tau has 6 isoforms A-F [23].

^†^Molecular weight (MW) in kDa, Residues is the number of amino acids [36]

After mTBI, biomarkers can potential diffuse through the extracellular space of the brain to penetrating blood vessels and enter the blood if the blood brain barrier is disrupted. Disruption of the blood brain barrier occurs with moderate or severe brain injury in about 40% of the cases [38, 39]. It is less certain whether disruption of the blood brain barrier occurs routinely with mild traumatic brain injury [13]. Using meningeal enhancement on MRI as a surrogate for disruption of the blood brain barrier, it has been suggested that transient disruption of the blood brain occurs in about 50% of the cases of mTBI [40]. It is possible that some biomarkers reach the blood by diffusing to the vessel walls in the brain and crossing the blood brain barrier directly [13]. Nonetheless, given that biomarkers can be detected in the blood of normal individuals with intact blood brain barriers and that many subjects with mTBI do not have a disruption of the blood brain barrier, it is likely that other routes account for some entry of biomarker into the blood after mTBI.

After TBI, levels of biomarkers in the cerebrospinal fluid rise rapidly, suggesting that these biomarkers have easy access to the cerebrospinal fluid from the interstitial fluid. Biomarkers can reach the cerebrospinal fluid by trans-ependymal flow into the ventricles or trans-pial flow into the subarachnoid space [35]. Once in the cerebrospinal fluid, biomarkers circulate by convection or diffusion. The traditional model of the cerebrospinal fluid circulation [41, 42] involves the production of cerebrospinal fluid by the choroid plexus, passage through the aqueduct to the fourth ventricle and then flow outward to the convexities of the brain and to the lumbar subarachnoid space [43].

The cerebrospinal fluid volume (150 ml) turns over four times per day [43]. Some cerebrospinal fluid is absorbed into the blood at the arachnoid granulations along the superior sagittal sinus. It is uncertain to what extent protein biomarkers can traverse the narrow passages of the arachnoid granulations to leave the cerebrospinal fluid and enter the blood. Most cerebrospinal fluid exits the cerebrospinal fluid compartment via lymphatic channels [44] at the cribriform plate (olfactory nerve), along other cranial nerve roots and along spinal roots. Radioactively labeled biomarker injected into the lateral ventricles appears in the lymph nodes within 6-8 hrs [44, 45], supporting the importance of these lymphatic routes for the absorption of cerebrospinal fluid and clearance of biomarkers from the cerebrospinal fluid.

It has been recognized for more than 25 years that radioactively labeled albumin injected into the interstitial fluid of the brain can be recovered from cervical lymph nodes draining the head [44]. Since albumin is larger in size than the protein biomarkers for TBI, it is likely that biomarkers released into the interstitial fluid have access to the blood through the lymphatic system. Two competing hypotheses have emerged as to the route of proteins from the interstitial fluid of the brain to the lymphatic system. One group has proposed that proteins and other waste products reach the lymph system through an intramural peri-arterial drainage system [46–50]. This model emphasizes intramural periarterial routes for drainage of protein molecules from the interstitial fluid. A second group [34, 51–57] has proposed a glymphatic system for removal of waste products from the interstitial fluid and their drainage into the lymphatic system. In their model, a convective flow of fluid through the interstitial space between small arteries and veins fosters drainage of waste and proteins into a para-venous space to the lymphatic system. The exact mechanism and precise anatomy by which biomarkers exit the interstitial fluid and drain into the lymph system and back to the blood remains unsettled [58, 59]. The relative contributions to blood biomarkers from biomarker transiting directly across the blood brain barrier versus transiting to the blood via the lymphatics is also unsettled [13].

Once in the blood, biomarkers must have a mode of exit. Little is known with certainty about the mode of elimination of biomarkers from the blood. Elimination could occur by redistribution to other compartments, renal excretion, hepatic metabolism, or intravascular proteolysis [13]. Dadas et al. ([60–62] have suggested that elimination of biomarkers after TBI is likely renal and that the rate of elimination is inversely proportional to biomarker molecular weight, with smaller biomarkers eliminated more rapidly than larger ones (e.g. S100B is eliminated more quickly than GFAP). An inverse relationship between renal elimination and molecular weight up to molecules as large as 70 kDa is known [63].

### What are the kinetics of blood biomarkers after mTBI?

**Fig. 2:**
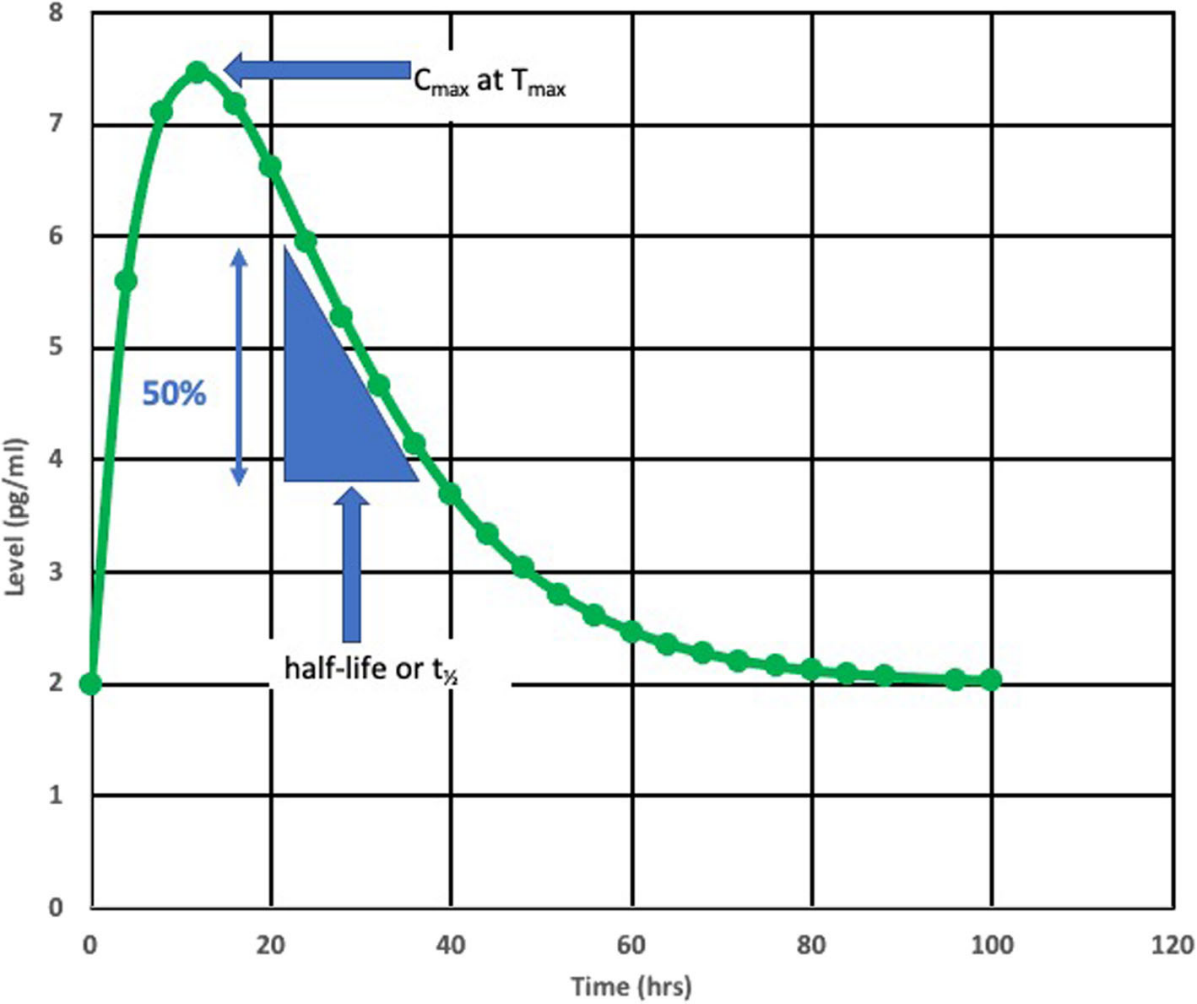
*T*_*max*_ is the time at which the biomarker is at its highest level. The half-life is the time needed for the biomarker level to drop by 50% during the elimination phase. Accurate estimates of half-life can only be made after the absorption phase is complete. With delayed or continuing absorption of biomarker after mTBI estimates of half-life are difficult

**Fig. 3:**
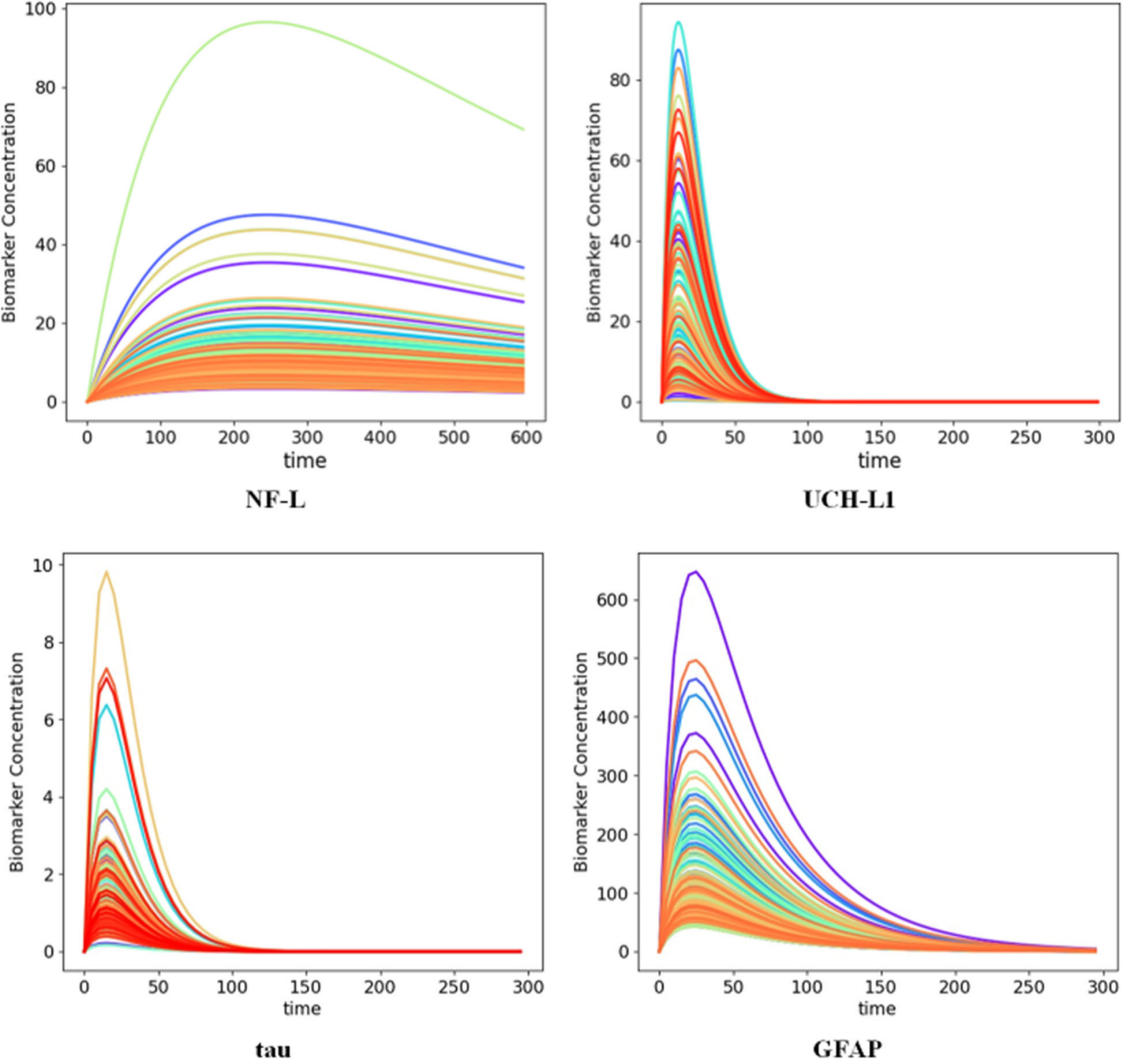
Calculated time-concentration curves for four blood biomarkers after mTBI based on a one-compartment pharmacokinetic model [74]. Each curve reflects a subject from the CARE dataset [31] who sustained a sports related concussion. Four blood biomarker levels were used to estimate the amount of biomarker released at impact and the pharmacokinetic model was used to generate the time-concentration curve based on Equation 3. Tests of modality suggested that biomarker levels were bi-modal [29]. Reproduced from [74] with permission

When the blood levels of biomarkers are sampled repeatedly after a mTBI, time-concentration curves can be created [64]. The descriptive kinetic parameters time to maximum concentration (*T*_*max*_), maximum concentration (*C*_*max*_), and half-life ($t_{\frac {1}{2}}$) are useful for describing these curves (Fig. 2). A limited number of formal kinetic studies of blood biomarkers after TBI are available [22, 60, 62, 65, 66]. Published time-concentration curves allow estimates of *T*_*max*_ and half-life for the commonly investigated blood biomarkers (Table 2) [1, 9, 11, 12, 17, 65]. Estimates of *T*_*max*_ and half-life are notably uncertain for NF-L due to a paucity of kinetic studies at longer time intervals after mTBI (Table 2). Azizi et al. [74] have modeled the blood levels of biomarkers after mTBI as a one-compartment pharmacokinetic model. In a one-compartment pharmacokinetic model, two first-order rate constants *k*_*a*_ (the absorption rate constant) and *k*_*e*_ (the elimination rate constant) determine the blood biomarker level at any time *t* after a mTBI (Eq. 3). Based on published values of *T*_*max*_ and half-life, Azizi et al. estimated *k*_*a*_ and *k*_*e*_ using Eqs. 1 and 2 [74]. Estimates for *k*_*a*_ and *k*_*e*_ are shown in Table 2. Kinetic modeling allows an estimate of *C*_*p*_ (the biomarker blood level at time *t*) if estimates are available for amount of biomarker released (*D*_0_), the fractional absorption rate (*F*), and the volume of distribution (*V*_*d*_) as illustrated in Fig. 3. (For details of the model see [74]).

1 $$ t_{\frac{1}{2}} = \frac {0.693}{k_{e}}  $$

**Table 2:** Kinetic parameters of commonly investigated biomarkers for mTBI

Biomarker	Normal Plasma	Normal CSF	Half-life	*T*_*max*_	*k*_*a*_‡	*k*_*e*_‡	References
	level (pg/ml)	level (pg/ml)	hrs∗	hrs∗			
GFAP	60-70	2100	36	24	0.080	0.019	[9, 11, 12, 17, 65]
NF-L	6-20	400-1100	500 ??	100-300 ??	0.009	0.001	[8, 12, 67, 68]
S100B	50-60	1800	1.5†	2	0.500	0.462	[9, 11, 27, 28, 65, 69, 70]
tau	1-5	270	10	8	0.070	0.069	[8, 11, 71, 72]
UCH-L1	8-10	4500	8	8	0.090	0.086	[9, 11, 17, 22, 65, 73]

^∗^Values for half-life and *T*_*max*_ are mid-range of reported estimates

^†^S100B undergoes rapid redistribution to other compartments before renal elimination

^??^Estimates are uncertain

^‡^Estimates for the absorption constant (*k*_*a*_) and elimination constant (*k*_*e*_) compartment pharmacokinetic model with first order kinetics from [74].

2 $$ T_{max} = \frac {\ln \left(\frac {k_{a}}{k_{e}}\right)}{k_{a}-k_{e}}  $$

3 $$ C_{p} = \frac{F*D_{0}*k_{a}}{V_{d} *(k_{a}-k_{e})} * \left(e^{-k_{e}t} -e^{-k_{a}t}\right)  $$

Azizi et al. [74] have suggested that the amount of biomarker released at impact (*D*_0_) could serve as a more reliable marker for TBI severity than an isolated blood biomarker level (*C*_*p*_) at a random time *t*. McDonald et al. [13] have emphasized that whether biomarker is released all-at-once at impact or is released in a delayed or continuing manner after mTBI is another known unknown. The delayed release of biomarker can be modeled in a one-compartment kinetic model by increasing *T*_*max*_ and decreasing the absorption rate constant (*k*_*a*_) while keeping the amount of biomarker released constant (*D*_0_) [74]. Furthermore, differing kinetic curves for each biomarker (Fig. 3) have implications for selecting the preferred sampling time for each biomarker.

### What is the optimal timing for blood biomarker sampling?

For some neurological diseases such as fronto-temporal dementia, Alzheimer disease, and multiple sclerosis, blood sample timing for biomarkers is unlikely to be critical [75–77]. With mTBI, like stroke [78], the timing of sampling is important. If blood biomarkers are sampled too early or too late compared to the *T*_*max*_, important elevations may be missed. Based on their approximate *T*_*max*_ (Table 2), it is predicted that S100B is best sampled between 0-4 hrs, UCH-L1 and tau between 4-12 hrs, GFAP between 12-36 hrs, and NF-L between 100-300 hrs. Estimates for the *T*_*max*_ of NF-L are not reliable and the actual value of the *T*_*max*_ could be much longer [12, 68]. Bogoslovsky et al. [10] have emphasized “intended context of use” for blood biomarkers and comment that blood sample times often depend upon the clinical setting (playing field, ED, hospital ward, or rehabilitation center) or the intended use (diagnosis of concussion, decisions around return to sport, or decisions around CT scanning).

Empirical studies are needed to confirm the best sampling times for blood biomarkers after mTBI depending on the intended context of use. In general, the best test performance, as measured by AUROC (area under the receiver operator curve), for S100B, UCH-L1, and tau occurs with early sampling times of 0-6 hrs (Table 3). Note that with the comparison of AUROCs across studies, there are no absolute standards for using the AUROC to evaluate the predictive capacity of a diagnostic test. An AUROC of 0.80 or greater generally suggests that the test is clinically useful [88]. Unlike specificity and sensitivity, AUROC provides a measure of test power that does not depend upon selecting a specific cutpoint [88]. Few studies have examined whether it is possible to sample blood too early after a mTBI. Even rapidly absorbed biomarkers, such as S100B, take a measurable amount of time to be released from injured neural elements, traverse the interstitial fluid, and cross the blood brain barrier or enter drainage pathways to the blood. On the other hand, the fall-off in levels with later sampling times is likely more pronounced for S100B than with UCH-L1 or tau. Due to its rapid clearance from the blood, sampling times after 6 hrs are likely to miss important elevations of S100B after mTBI. With the longer half-life of NF-L, a later sampling time can yield a good AUROC. It is unclear if there is a penalty for sampling NF-L too early after a mTBI (Table 3). GFAP, which has a longer half-life than UCH-L1, S100B, or tau, may be better sampled later than the 0-6 hr time frame. For example, Welch et al. [65] found that GFAP as a predictor of CT positivity performed better when drawn at 12-18 hrs instead of 0-6 hrs. More investigation is needed to set the optimal sampling time for each blood biomarker for mTBI.

**Table 3:** Performance at different sampling times for studies with multiple sampling intervals (Area under receiver operator curve)†

Biomarker	Study	Year	Use	0-1	0-6	6-24	24-48	48-144	>144	
GFAP	Asken [79]	2018	concussion		0.63	**0.71**				
	McCrea [31]	2020	concussion		0.68		0.57			
	Meier [80]	2017	concussion		0.53		**0.56**			
	Papa [17]	2019	concussion	0.76	0.76	0.8	0.87	0.89		
	Posti [81]	2017	concussion			**0.63**	0.37			
	Yue [82]	2019	CT-/MRI+		0.72	**0.85**				
	Welch [65]	2017	CT+/CT-		0.84	**0.94**				
	McDonald [68]	2021	concussion				0.60	0.52	0.54	
	Giza [83]	2021	concussion		0.75		0.63			
NF-L	McCrea [31]	2020	concussion		**0.56**		0.49			
	Shahim [84]	2017	PCS		0.82		0.73	**0.83**	0.66	
	Shahim [72]	2018	RTS		**0.82**		0.72	0.73	0.73	
	McDonald [68]	2021	concussion				0.73	0.85	0.79	
	Giza [83]	2021	concussion		0.75		0.63			
S100B	Asken [79]	2018	concussion		0.75	0.69				
	Meier [80]	2017	concussion		0.72		0.58			
	Welch [65]	2017	CT+/CT-		**0.78**	0.75				
	Shahim [85]	2014	PCS		0.68			0.55		
	Rogatzki [86]	2021	concussion	0.48	0.24					
tau-T	Asken [79]	2018	concussion		**0.74**	0.62				
	McCrea [31]	2020	concussion		**0.55**		0.31			
	Shahim [85]	2014	PCS		0.90			0.76		
	Gill [87]	2017	RTS		**0.80**			0.74		
	Shahim [72]	2018	RTS		0.67		0.57	0.52		
	McDonald [68]	2021	concussion				0.51	0.55	0.72	
	Giza [83]	2021	concussion		0.50		0.46			
UCH-L1	Asken [79]	2018	concussion		**0.62**	0.60				
	McCrea [31]	2020	concussion		**0.66**				0.51	
	Meier [80]	2017	concussion		0.74				0.55	
	Papa [17]	2019	concussion	0.65	0.65	0.77	0.74	0.50		
	Posti [81]	2017	concussion		**0.52**	0.46				
	McDonald [68]	2021	concussion				0.61	0.56	0.57	
	Giza [83]	2021	concussion		0.73		0.62			

^∗^Use: concussion versus controls, mTBI with CT+ versus CT-, delayed return to sport versus no delay, CT- and MRI+ versus CT- and MRI-, post-concussion syndrome versus no post-concussion syndrome.

^†^Time intervals are in hours and are approximate across studies.

**Bolded AUROC** is the highest row value.

Values are rounded to 2 decimals. No tests of significance were applied.

### What are the confounding factors for blood biomarker levels after mTBI?

One confounding factor for biomarker blood levels is total blood volume (TBV). Individuals with higher blood volumes have lower blood biomarker levels. Manouchehrinia et al. [89] studied NF-L levels in 2,586 multiple sclerosis patients and 662 controls. Mean NF-L levels were 7.52 pg/ml in the controls and 11.68 pg/ml in the multiple sclerosis cases. Plasma NF-L levels dropped by -0.15 pg/ml for each liter of TBV in controls and -0.17 pg/ml per liter of TBV in multiple sclerosis patients.

Another confounding factor is renal function. Since some elimination of blood biomarker is likely renal, impaired renal function can lengthen biomarker half-life in the blood and elevate biomarker blood levels. Akamine et al. [90] investigated the relationships between blood NF-L levels and renal function in 43 healthy adults and 188 patients with diabetes mellitus. Blood neurofilament levels correlated significantly with creatinine levels in healthy controls (r=+0.50) and diabetic subjects (r=+0.56).

Endogenous levels of plasma neurofilament protein, tau, and GFAP are known to rise with age [91–93]. In the TRACK-TBI study, Gardner et al. [94] found that the ability of GFAP to discriminate between CT+ and CT- subjects was less in the older age group (AUROC=0.73) than the middle age (AUROC=0.92) or young age group (AUROC=0.93). This age-related effect was not noted with tau. Ward et al. [95] retrospectively examined sensitivity and specificity of the Banyan Brain Trauma Indicator (a tandem GFAP and UCH-L1 assay). They found that while sensitivity was 100% in the ≥65 and <65 age groups, specificity dropped from 0.44 to 0.13 in the older age group. They attributed this drop in specificity to higher levels of GFAP and UCH-L1 in the older CT- subjects. Levels of GFAP and UCH-L1 were not significantly higher by age in the CT+ subjects. Iverson et al. [96] found both older patients with uncomplicated mTBI and orthopedic controls had higher NF-L levels when blood was drawn within 19 hours of injury. Calcagnile et al. [97] found higher levels of S100B in older patients with mTBI and found that the specificity of the test to identify CT+ patients was reduced in the elderly.

### How long do blood biomarker levels remain elevated after mTBI?

A few studies have examined biomarker levels months or years after mTBI. Blood half-lives of most biomarkers (UCH-L1, S100B, GFAP) are short (less than 48 hrs) [12]. With the exception of NF-L which has a longer half-life, biomarker levels at 30 to 90 days are expected to be normal unless there is ongoing neural degeneration or re-injury. Shahim et al. [98] followed 89 subjects with mTBI for up to five years. In some subjects, they found elevated NF-L levels up to 5 years after mTBI. Levels of GFAP were elevated for up to 30 days after mTBI. Tau and UCH-L1 were at baseline levels after 30 days [98]. Shahim et al. [99] found elevated NF-L levels in the cerebrospinal fluid of professional hockey players with post-concussion syndrome for up to one year after mTBI. Pattinson et al. [100] studied 109 military personnel with a past history of traumatic brain injury. They found TBI subjects did not differ from controls in levels of tau, NF-L, *Aβ*40, or *Aβ*42. McDonald et al. [68] found elevated blood NF-L levels for up to 13 days after concussion in Australian football players and these levels discriminated concussed players from control players. Bogoslovsky et al. [101] measured levels of amyloid- *β*42, tau, and GFAP at 0, 30, and 90 days after TBI in 34 subjects entered into the COBRIT trial. Subjects were of mixed TBI severity; none had simple concussions. They found elevated levels of three biomarkers compared to controls, although levels of tau and GFAP were dropping. Currently, explanation for persistently elevated biomarker levels after mTBI in some subjects remains uncertain. Persistent elevations of NF-L after mTBI could reflect ongoing release of biomarker from original injury or re-injury [13]. The prognostic significance of these late elevations is uncertain. Upregulation of protein synthesis in some patients after TBI has been reported [102].

### Can blood biomarkers diagnose concussive or sub-concussive hits to the head?

Studies have shown that blood biomarkers have a modest capability to distinguish healthy controls from concussed subjects (Table 4). Most of the areas under the receiver operator curves (AUROC) are below 0.80, suggesting only fair discrimination between healthy controls and concussed subjects. Papa et al. [17] found higher levels of UCH-L1 (AUROC =0.65) and GFAP (AUROC= 0.76) in concussed head injury subjects compared to head injury controls without concussion symptoms at 0 to 4 hrs. In the 0 to 6 hr time frame, McCrea et al. [31] found only modest values for the AUROC for UCH-L1 (0.66), tau (0.55), NF-L (0.56), and GFAP (0.68). One explanation for these low AUROC levels may be that the response to a concussion is bi-modal, with most subjects showing a modest rise in blood biomarker levels and a smaller subset showing a more robust rise in biomarker levels. Bui et al. [29] in their review of data from the CARE mTBI dataset [30, 31] that biomarker levels may be bimodal after mTBI, with only a minority of concussed subjects exhibiting large rises in biomarker levels.

**Table 4:** Area under the receiver operator curve (AUROC) for diagnosis of concussion per biomarker

Marker	Study	Year	Study Size	Sampling	AUROC∗
	Lead Author		(N)	Time (hrs)†	
GFAP	Gill [71]	2018	277	0-48	**0.93**
	Papa [17]	2019	452	0-4	0.76
	Giza [83]	2021	167	0-6	0.75
	Lewis [103]	2017	188	0-6	0.70
	McCrea [31]	2020	206	0-6	0.68
	Posti [81]	2017	44	0-24	0.63
	Asken [79]	2018	29	0-4	0.63
	McDonald [68]	2021	28	2 days	0.60
	Meier [80]	2017	32	0-6	0.53
NF-L	McDonald [68]	2021	20‡	6 days	0.85
	Gill [71]	2018	277	0-48	0.77
	Giza [83]	2021	167	0-6	0.73
	McCrea [31]	2020	206	0-6	0.56
	Wallace [104]	2018	12	6 days	NS
S100B	Asken [79]	2018	29	0-4	0.75
	Meier [80]	2017	32	0-6	0.72
	Lewis [103]	2017	188	0-6	0.69
	Shahim [85]	2014	35	0-1	0.67
	Rogatzi [86]	2021	8	0-1	0.48
tau-T	Shahim [85]	2014	35	0-1	**0.86**
	Asken [79]	2018	27	0-4	0.74
	McDonald [68]	2021	20‡	13 days	0.72
	Gill [71]	2018	277	0-48	0.68
	McCrea [31]	2020	206	0-6	0.55
	Giza [83]	2021	167	0-6	0.50
	Wallace [104]	2018	11	6 days	NS
	Wallace [104]	2019	11	14 days	NS
UCH-L1	Meier [80]	2017	32	0-6	0.74
	Giza [83]	2021	167	0-6	0.73
	McCrea [31]	2020	206	0-6	0.66
	Lewis [103]	2017	188	0-6	0.65
	Papa [17]	2019	452	0-4	0.65
	Asken [79]	2018	26	0-4	0.62
	McDonald [68]	2021	20‡	2 days	0.61
	Posti [81]	2017	44	0-24	0.52

^∗^The bolded AUROC value are ≥**0.80**.

^†^Times are in hours except where noted.

^‡^Concussed male football players only, NS for 8 females

Some studies have shown that sub-concussive hits to the head can elevate biomarker levels. For example, Kawata et al. [105] studied S100B levels before and after college football practices. None of the subjects sustained a concussion. They compared high-impact to low-impact subjects. Subjects with more hits and hits with higher peak linear and rotational accelerations showed higher levels of S100B. This pattern continued over multiple practices. However, there was no cumulative effect on S100B levels over the season. On the other hand, Hulbregtse et al. [2020], in a controlled randomized trial, compared soccer players who headed or kicked a soccer ball, found no rise in plasma S100B levels at 0, 2, or 24 hrs after 10 headers. Puvenna et al. [14] found that the number of sub-concussive hits during a varsity college football game correlated with S100B levels but not UCH-L1 levels. Over the course of a football season, sub-concussive hits to the head caused no persistent increase in blood tau biomarker levels [106].

### Are blood biomarkers predictive of CT scan positivity after mTBI?

Concussed patients with abnormalities on either MRI or CT scan are termed complicated mTBI [107]. In the CENTER-TBI project, 42.3% of 2955 subjects with mTBI were classified as complicated mTBI. One goal of blood biomarker research has been to discriminate between patients with abnormalities on CT scan (CT positive) from subjects without CT scan abnormalities (CT negative). The intent is to identify mTBI patients who are unlikely to benefit from CT scanning. In general, the biomarkers S100B and GFAP have shown a moderate ability to discriminate between CT-positive and CT-negative subjects (Table 5).

**Table 5:** Area under the receiver operator curve (AUROC) for CT positivity by biomarker

Marker	Study	Year	Study Size	Sampling	AUROC
	Lead Author		(N)	Time (hrs)	
GFAP	Czeiter [108]	2020	2867‡	0-24	**0.89**
	Gardner [94]	2018	169	0-24	**0.88**
	Huebschmann [109]	2020	121	0-12	**0.81**
	Welch [65]	2016	251	0-6	0.78
	Gill [71]	2018	277	0-48	0.77
	Yue [82]∗	2019	450	0-24	0.77
	Lewis [103]	2017	188	0-6	0.66
NF-L	Gill [71]	2018	277	0-48	0.65
S100B	Welch [65]	2016	251	0-6	0.75
	Allouchery [110]	2018	1449	0-2	0.72
	Jones [111]	2020	679	0-6	0.69
	Egea-Guerrero [112]	2018	260	0-6	0.67
	Lewis [103]	2017	188	0-6	0.65
	Yue [82]∗	2019	450	0-24	0.56
tau-P	Gardner [94]	2018	169	0-24	**0.93**
tau-T	Gardner [94]	2018	169	0-24	0.71
	Diaz-Arrastia [113]	2014	171	0-24	0.67
	Gill [71]	2018	277	0-48	0.66
UCH-L1	Welch [65]	2016	251	0-6	0.79
	Lewis [103]	2017	188	0-6	0.60
	Yue [82]∗	2019	450	0-24	0.59

The bolded AUROC values are ≥**0.80**

^∗^This study compared MR- vs. MRI+ in CT- subjects. All other studies are CT- vs. CT+.

^†^1951 of 2867 subjects had mTBI.

^‡^Concussed male football players only, females excluded.

Cost savings are possible if patients with mild head injuries who are unlikely to benefit from CT scanning are excluded from neuroimaging. In Europe, S100B has been extensively studied as a biomarker that could reduce the use of CT scanning for minor head injuries [26, 114]. If a cutpoint of 100 pg/ml is used for S100B within 6 hrs of head injury, the test is 97% sensitive and 34% specific in predicting subjects with CT scan abnormalities [26]. A risk occurs with S100B due to its short half-life of 1-2 hrs [26], making false-negative tests possible if blood sampling is delayed excessively. However, false-negative tests with S100B have only rarely been reported, and the biggest problem in the use of the test has been low specificity. The most recent validation of the Scandinavian Head Injury Guideline with a cutoff of 100 pg/ml of S100B within 6 hrs of mild head injury showed a sensitivity of 0.94 and a specificity of 0.19 in predicting abnormalities on CT scan. The lower specificity has been attributed to the greater age of the study group, with older patients having higher baseline levels of S100B [115]. If fully applied, the Scandinavian Guidelines with S100B testing would have reduced CT scanning by 9% [115]. Jones et al. [111] evaluated the predictive value of S100B for CT abnormalities in 679 mTBI subjects. Blood was drawn within 6 hours of head injury. With a cutoff of 100 pg/ml, they found a sensitivity of 0.85, a specificity of 0.34, a positive predictive value of 0.72, and a negative predictive value of 0.97 for abnormalities on the CT scan after mTBI. Calcagnile et al. [116] measured S100B blood levels within 3 hours of injury in 726 subjects with mTBI. Using a cutoff of 100 pg/ml, they found no subjects with a positive CT scan in the 229 subjects with S100B below the cutoff point and found a positive CT scan in 150 of the 497 subjects with an S100B over the cutoff point. Another study found an S100B level above 100 pg/ml within 6 hours of injury predictive of CT scan abnormalities [112]. In a meta-analysis of eight published studies, Oris et al. [117] found a pooled sensitivity of 100% and a pooled specificity of 35% when S100B levels were obtained within 3 hrs of injury. Cutoff values for S100B varied by study between 6 and 200 pg/ml. Ananthaharan et al. [118] monitored the use of the S100B Scandinavian CT scanning guidelines in mTBI. In 69 subjects with a mTBI and S100B level below the cutoff of 100 pg/ml, all had a negative CT scan.

In the United States, the FDA has approved the Banyan BTI™(Brain Trauma Indicator) to predict CT scan abnormalities after mTBI. Blood is sampled within 12 hrs of head injury. Test sensitivity is 97.5% and specificity is 36.5% on the FDA application [119]. The FDA has recently approved a handheld testing platform for GFAP and UCH-L1 levels with results available within 15 mins [120]. Okonkwo et al. [121] have reported on the point of care testing of 1359 traumatic brain injury subjects of all severity levels. The AUROC for prediction of a positive CT scan for GFAP (0.85) was significantly higher than S100B (0.67) for subjects evaluated within 24 hr of injury.

Gill et al. [71] studied 277 subjects with suspected mTBI in the emergency room setting. Levels of UCH-L1, NF-L, tau, and GFAP were measured by single-molecule array assay. By AUROC, GFAP outperformed tau and NF-L in predicting neuroimaging abnormalities (CT or MRI). Another study, done with the less sensitive ELISA assay, suggested that GFAP was a better predictor of CT scan abnormalities than S100B [122]. The TRACK-TBI study evaluated 450 mTBI subjects who were CT scan negative. GFAP levels were higher in MRI positive mTBI subjects than MRI negative subjects. Both groups of mTBI were significantly higher than orthopedic controls or healthy controls [82]. Another study confirmed the ability of GFAP to discriminate between CT positive and CT negative subjects with mTBI [109]. Lewis et al. [103] evaluated the ability of GFAP, UCH-L1, and S100B to stratify 247 emergency room patients who had biomarker levels within 6 hrs of injury. All three biomarkers were higher in subjects with complicated mTBI than mTBI and higher in mTBI than no TBI. A cutoff of 30 pg/ml for GFAP, 30 pg/ml for UCH-L1, and 35 pg/ml for S100B gave a sensitivity of 44% for GFAP, 95% for UCH-L1, and 96% for S100B in distinguishing between no TBI and mTBI. Cutoffs for distinguishing between complicated mTBI and mTBI were not provided.

### Are blood biomarkers predictive of severity and outcome after mTBI?

Several clinical measures have been used to assess the clinical severity of mTBI including the SAC (Standardized Assessment of Concussion), the SCAT (Sports Concussion Assessment Tool), the BESS (Balance Error Scoring System), BSI-18 (Brief Symptom Inventory-18), and the ImPACT (Immediate Post-Concussion Assessment and Cognitive Test (ImPACT). In general, few correlations have been found between these clinical measures and blood biomarker levels after mild TBI [29, 31, 68, 79, 83]. Giza et al. [83] found a weak correlation (r= 0.36) between acute GFAP levels and the BESS. Bui et al. [29] examined clustering of biomarker trajectories for tau, UCH-L1, GFAP, and NF-L. In general biomarker trajectory did not predict TBI severity with three exceptions: clusters with higher trajectories of NF-L and GFAP did worse on the SAC at 6 hrs post mTBI, and a cluster with higher UCH-L1 did worse on the verbal memory test at 7 days after post return to sport. Nonetheless, the limited capacity of blood biomarkers to correlate with mTBI severity has been disappointing to date.

The prediction of outcome after mTBI has been difficult, whether the outcome is measured by the time to recovery, the persistence of symptoms, or the development of a post-concussion syndrome (PCS). Accurate models to predict outcome from mTBI are not available [123]. As assessed by AUROC, blood biomarkers are weakly predictive of outcome after mTBI (Table 6). Both NF-L and tau have shown a limited predictive ability for return to sport in professional hockey players after concussion [67, 72, 84, 85]. NF-L levels measured up to 144 hours after injury and tau measured up to 12 hours after injury were predictive of return to sport [71, 72, 85]. A small study did not find cleaved tau levels predictive of post-concussion syndrome [127]. Gill et al. [87] found higher tau levels in student athletes with a prolonged return to sport after sports-related concussion. In a larger study, Pattinson et al. [124] studied 127 concussed student athletes and found that early tau levels were weakly predictive of return to sport. Similarly, Hossain et al. [126] found a weak correlation between admission tau levels and Glasgow Outcome Scale-Extended score at 6-12 months.

**Table 6:** Area under the receiver operator curve (AUROC) for biomarker studies predicting outcome from mTBI

Marker	Study	Year	N	Time (hrs)	AUROC	Outcome
						Predicted
GFAP	Huebschmann [109]	2020	121	0-12	0.69	GOS-E >6
	Pattinson [124]	2020	59	0-12	0.40	RTS <8 days
NF-L	Shahim [72]	2018	87	0-1	0.83	RTS <15 days
	Shahim [84]	2017	35	0-1	0.82	RTS <6 days
S100B	Shahim [85]	2014	28	0-1	0.68	Duration of PCS <6 days
	Babcock [125]	2008	76	0-6	0.47	Onset of PCS
tau-A	Shahim [67]	2016	28	0-1	**0.87**	RTS <10 days
tau-C	Shahim [67]	2016	28	0-1	0.58	RTS <10 days
tau-T	Shahim [85]	2014	28	0-1	**0.91**	Duration of PCS <6 days
	Gill [87]	2017	43	0-6	0.81	RTS <10 days
	Pattinson [124]	2020	59	Recovered	0.75	RTS <8 days
	Shahim [72]	2018	87	0-1	0.67	RTS <15 days
	Pattinson [124]	2020	55	24-48	0.66	RTS <8 days
	Hossain [126]	2020	105	0-24	0.56	GOS-E >7

*RTS* Return to sport.

bolded AUROC values ≥**0.80**

## Conclusions

This review examined eight unresolved issues related to the use of blood biomarkers for the diagnosis and management of mTBI. A number of review limitations should be emphasized. We focused on the five most commonly investigated blood biomarkers after mTBI and did not address other blood biomarkers such as neuron specific enolase (NSE), myelin basic protein, inflammatory biomarkers, or microRNA [128]. Our review methodology was a focused and not systematic. We have used the more general term blood biomarker level and have not distinguished between plasma and serum levels (although plasma and serum levels differ, they are generally highly correlated [129]). Analytic issues such as test-retest reliability, test sensitivity, inconsistencies across test platforms, and issues related to storage and handling of samples have not been addressed in this work (for a discussion of some of these issues see [13]. We focused on blood levels of biomarkers and did not address biomarker levels in the cerebrospinal fluid, urine, or saliva [130, 131].

Evidence from the clinical setting and from animal models indicates a disruption of the blood brain barrier occurs after moderate to severe traumatic brain injury [132–136, 136–139] and that biomarkers can cross the blood brain barrier into the blood [11, 62]. In mTBI, the blood brain barrier may be disrupted as well [132, 137]. Nonetheless, a disruption of the blood brain barrier may not be essential for the entry of biomarkers into the blood. Opinion is growing that protein biomarkers enter the blood by alternate routes [15] including the intramural periarterial drainage system and the glymphatic system. The route of entry by biomarkers, whether by crossing the blood brain barrier, through glymphatics, or via other drainage pathways, likely influences the rate of absorption and kinetics of blood biomarkers for mTBI.

Kinetic parameters such as *T*_*max*_ and half-life provide important information about the behavior of mTBI biomarkers in the blood. Current estimates of these kinetic parameters are imprecise [13]. Better estimates of kinetic parameters would allow more precise modeling of biomarker levels after TBI and foster more precise cutoff levels and sampling times. Available evidence suggests that the optimal sampling time for S100B after mTBI is between 1 and 3 hours, for tau and UCH-L1 optimal sampling time is likely between 2 and 8 hours. The optimal sampling time for GFAP is likely later, possibly between 6 and 18 hours. There is less certainty about the optimal sampling time for NF-L due to uncertainty about biomarker half-live and T _*max*_, but it is likely later than the other commonly measured biomarkers for mTBI. Several confounding factors for blood biomarker levels have been identified including increasing age, renal failure, and increased blood volume. However, corrections in biomarker levels are not routinely made for these confounding factors. Elevated levels of both tau and NF-L have been observed months or years after traumatic brain injury. The significance of these persistent elevations in blood biomarker levels is uncertain.

Blood biomarkers have a modest ability to detect sub-concussive head injuries and a modest ability to discriminate between concussed and non-concussed subjects. Some subjects with verified concussions may have minimal increases in blood biomarker levels which overlap with the levels found in control subjects. None of the studied blood biomarkers have been shown to reliably correlate with repetitive sub-concussive hits or cumulative concussive hits to the head. Blood biomarkers have had some success in predicting CT scan positivity after mild traumatic brain injury. S100B is widely used in Europe, and the GFAP/UCH-L1 tandem test has been approved for use in the United States. Despite impressive levels of sensitivity, these tests lack high levels of specificity. Correlations between blood biomarker levels and mTBI severity have been disappointing to date. Evidence is inconclusive as to whether any blood biomarker predicts recovery time, post-concussion syndrome, or return to sport after mild traumatic brain injury. Both tau and neurofilament light chain protein have shown limited promise for this application. Return to sport and return to work decisions are influenced by complex educational, sociological, and psychological factors not captured in a blood biomarker test [140].

## Data Availability

Not applicable.

## Abbreviations

AUROC: Area under receiver operator curve
CARE: Concussion Assessment, Research, and Education Consortium Study
CENTER-TBI: Collaborative European NeuroTrauma Effectiveness Research in Traumatic Brain Injury
CT-: CT negative (without findings)
CT+: CT positive (with findings)
GFAP: glial acidic fibrillary protein
NF-L: neurofilament light chain
PCS: post-concussion syndrome
RTS: Return to sport
tau-C: cleaved tau
tau-P: phosphorylated tau
tau-T: total tau
TBI traumatic brain injury; UCH-L1: ubiquitin C-terminal hydrolase-L1
